# Sex Differences in Outcomes among Stroke Survivors with Non-Valvular Atrial Fibrillation in China

**DOI:** 10.3389/fneur.2017.00166

**Published:** 2017-04-27

**Authors:** Yan Hong, Xun Yang, Wenjuan Zhao, Xianghui Zhang, Junli Zhao, Yuanju Yang, Xianjia Ning, Jinghua Wang, Zhongping An

**Affiliations:** ^1^Department of Neurology, Tianjin Huanhu Hospital, Tianjin, China; ^2^Tianjin Key Laboratory of Cerebral Vascular and Neurodegenerative Disease, Tianjin, China; ^3^Department of Epidemiology, Tianjin Neurological Institute, Tianjin, China; ^4^Department of Neurology, Tianjin Medical University General Hospital, Tianjin, China

**Keywords:** sex differences, acute ischemic stroke, non-valvular atrial fibrillation, outcome, risk factors, China

## Abstract

Atrial fibrillation (AF) significantly increases the risk of stroke and disease burden and is an established predictor of poor outcomes after stroke. However, data regarding sex differences in long-term outcomes following stroke in patients with AF are scarce. We thus aimed to assess these differences. We recruited 951 consecutive patients with acute ischemic stroke and non-valvular atrial fibrillation (NVAF) treated at three hospitals in Tianjin, China, from January 2006 to September 2014. Information regarding stroke subtype, severity, risk factors, and outcomes (mortality, dependency, and recurrence) at 3, 12, and 36 months after stroke was recorded. The prevalence of NVAF was 8.4% overall, with a higher frequency in women than in men (11.3 vs. 6.9%, *P* < 0.001). Among patients with NVAF, women were older than men. Women were more likely than men to have severe stroke (38.8 vs. 29.5%, *P* < 0.001), high levels of total cholesterol and high- and low-density lipoprotein cholesterol (all *P* < 0.001), hypertension (69.1 vs. 61.2%, *P* = 0.012), dyslipidemia (29.8 vs. 20.7%, *P* = 0.001), and obesity (18.5 vs. 11.6%, *P* = 0.003); they were less likely than men to be current smokers (12.2 vs. 33.6%, *P* < 0.001) and to consume alcohol (0.9 vs. 13.9%, *P* < 0.001). There were greater risks of dependency and recurrence at 36 months after stroke in women than in men [odds ratios (95% confidence intervals), 1.64 (1.02–2.64) for dependency, *P* = 0.043; and 2.03 (1.28–3.20) for recurrence, *P* = 0.002] after adjustment for stroke subtype, severity, and risk factors. These findings suggest that it is crucial to emphasize the need for individualized stroke prevention education and promotion of healthy lifestyles in order to improve NVAF-related stroke outcomes and reduce disease burden in women.

## Introduction

Atrial fibrillation (AF) is the most common sustained cardiac arrhythmia. The prevalence of AF in the general population in Western countries is reported to range from 1 to 2% (1–3), and it increases substantially with age (4). Patients with AF have a fivefold increased risk of stroke compared to that of the general population (5). Almost one-third of patients with first-ever stroke have a history of AF, which is related to a greater risk of neurological impairment, disability, increased recurrence, and more frequent dementia (6, 7).

With respect to sex differences in AF, women with AF have been shown to have an increased risk of cardiovascular events, including stroke (8, 9). Women in general have also been shown to have a greater incidence of stroke than do men (3 vs. 1.6%) (10–14). Previous studies have indicated higher mortality rates for persons after stroke with AF, with rates between 17 and 32.5% at 30 days after stroke (15–20) and between 30.5 and 63.0% at 12 months after stroke (15, 21–23). Moreover, worse neurological function has been reported to occur in persons after stroke with AF (24). However, sex differences in outcomes, including mortality, dependency, and recurrence after stroke, for patients with AF are controversial. Poor stroke outcomes have been found in women in some studies [e.g., Ref (24).], other studies have reported a greater risk of death at 1 year after stroke in men (15), while still others have reported no sex differences (20, 25). We, therefore, aimed to explore the sex differences in demographic characteristics, clinical features, previous histories of disease, and long-term outcomes after stroke in patients with AF.

## Materials and Methods

### Patient Selection

This study used data from a stroke registry from three hospitals in Tianjin, China (Tianjin Medical University General Hospital, Tianjin Huanhu Hospital, and Tianjin Haibin People’s Hospital). Inclusion criteria for patients with stroke were as described in a previous study (26). Briefly, from January 2006 to September 2014, we prospectively collected data on clinical characteristics and outcomes of all patients with ischemic stroke who were admitted to the stroke units in these three hospitals within 72 h after stroke onset. Stroke events were defined according to the World Health Organization’s criteria, and all cases of stroke were confirmed by computed tomography or magnetic resonance imaging, as required (27). Patients with transient ischemic attack were excluded from this study. All persons after stroke with non-valvular atrial fibrillation (NVAF) who had experienced stroke were analyzed in this study. NVAF was defined as a self-reported history of NVAF, from electrocardiography on admission showing NVAF confirmed by at least one electrocardiogram, or by the presence of arrhythmia during hospitalization.

### Clinical Features

Data collection and evaluation were performed by senior neurologists using standardized variable definitions and scores. Stroke subtypes, which were classified on admission, included total anterior circulation infarct, partial anterior circulation infarct (PACI), lacunar infarct, and posterior circulation infarct (POCI), according to the Oxfordshire Community Stroke Project (OCSP) criteria (28). Stroke severity was categorized into three groups according to the National Institutes of Health stroke scale (NIHSS): mild (NIHSS score: ≤7), moderate (NIHSS score: 8–16), and severe (NIHSS score: ≥17) (29). Simultaneously, the primary risk factors for stroke, including hypertension, diabetes mellitus (DM), and hyperlipidemia, were defined according to self-reported medical history, and obesity was defined as a body mass index ≥30 kg/m^2^. The NIHSS score and Barthel index (BI) were assessed on admission and at discharge, and the modified Rankin Scale (mRS) score was assessed on admission, at discharge, and at 3 and 12 months after stroke. Information of oral anticoagulants on discharge was recorded.

### Outcomes Definitions

Outcomes included mortality, dependency, and recurrence rates at 3, 12, and 36 months after stroke. Mortality was defined as all-cause cumulative deaths at each follow-up point. Dependency was defined as an mRS score >2 (30). Recurrence was defined as all new-onset vascular events, including stroke, myocardial infarction, and venous thrombosis. Follow-up was implemented according to a predetermined procedure; the same senior neurologist collected data at 3, 12, and 36 months after stroke. Follow-up for all patients occurred through face-to-face interviews and/or telephone calls.

To ensure data quality, three groups of senior trained neurologists (the assessment group, the follow-up group, and the quality control group) were responsible for determining the nervous system score at admission, for the reexamination (including of neurological score, risk factor management, and directing the treatment and rehabilitation) during follow-up, and a sampled confirmation of 20% of all patients each month, respectively.

### Statistical Analysis

Continuous variables, including age, NIHSS score, BI, mRS score, total cholesterol (TC), triglycerides (TGs), high-density lipoprotein cholesterol (HDL-C), low-density lipoprotein cholesterol (LDL-C), fasting glucose (FG), and HbA1c were presented as means (SD) or medians (interquartile range), as appropriate, and compared between men and women using the Student’s *t*-test or Mann–Whitney *U*-test. Dichotomous variables including stroke subtype, stroke severity, medical history, stroke risk factors, and outcomes at different periods after stroke were presented as numbers (percentages), and the sex differences in these risk factors were compared by the chi-squared test. A univariate analysis of sex differences in outcomes was performed with logistic regression models and presented as unadjusted odds ratios (ORs) with 95% confidence intervals (CIs). The multivariate analysis of sex differences in outcomes was performed with a logistic regression model with factors found to be statistically significant in the univariate analysis (including age, stroke subtype, severity, medical history, and risk factors) as covariates, and results were presented as adjusted ORs with 95% CIs. All statistical analyses were performed using SPSS version 15.0 (SPSS Inc., Chicago, IL, USA), and a two-tailed *P* < 0.05 indicated statistical significance.

## Results

**Figure 1:**
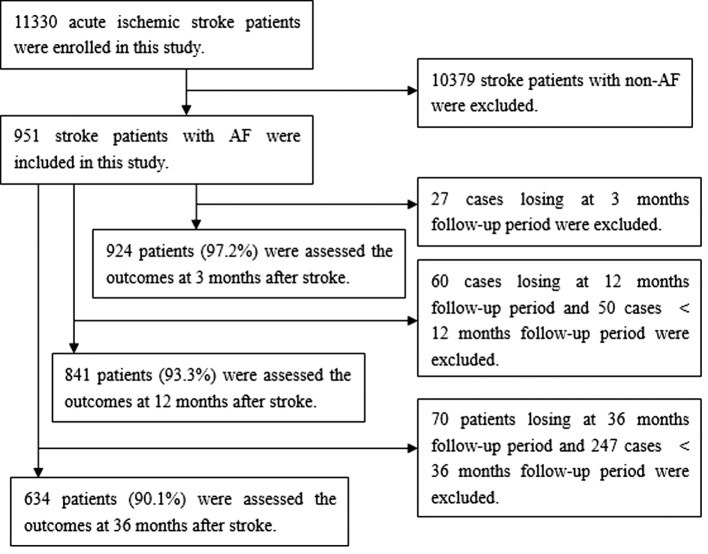
**Flow diagram of participants**. There were 924 cases of atrial fibrillation (AF) (97.2%) included for the outcomes analysis at 3 months poststroke after excluding 27 patients lost to follow-up; 841 cases (93.3%) included at 12 months poststroke after excluding 60 patients lost to follow-up and 50 cases <12 months follow-up period; and 634 cases (90.1%) included at 36 months poststroke after excluding 70 patients lost to follow-up and 247 cases <36 months follow-up period.

Overall, 11,330 consecutive patients with ischemic stroke were registered in the stroke units of the three hospitals in Tianjin during the study period. Of these, there were 951 patients (8.4%) with NVAF. There were 924 cases of NVAF (97.2%) included for outcome analysis within 3 months poststroke after excluding 27 patients lost to follow-up; 841 cases (93.3%) included within 12 months poststroke after excluding 60 patients lost to follow-up; and 634 cases (90.1%) included within 36 months poststroke after excluding 70 patients lost to follow-up (Figure 1).

### Sex Differences in Clinical Features in Patients with NVAF

The prevalence of NVAF in patients with stroke was higher in women than in men (11.3 vs. 6.9%, *P* < 0.001). Women were more likely than men to be older and had a greater frequency of severe stroke. The median NIHSS and mRS scores were higher in women, but the BI was lower than that in men (all *P* < 0.001). Similar frequency of oral anticoagulants on discharge was observed between men and women (76.4 vs. 76.9%, *P* > 0.05). TC, HDL-C, and LDL-C levels were higher in women than in men, but not the levels of TG, glucose, or glycosylated glucose. Moreover, the frequency of OCSP classifications was not significantly different between men and women (Table 1). However, different results were found for stroke survivors without AF. Women were more likely than men to have PACI. The levels of FG and glycosylated glucose were higher in women than in men (all *P* < 0.0001; Table S1 in Supplementary Material).

**Table 1:** **Sex differences in clinical and demographical characteristics in ischemic stroke patients with atrial fibrillation (AF)**.

Characteristics	Men	Women	*P*
Non-valvular atrial fibrillation prevalence, *n* (%)	518 (6.9)	433 (11.3)	<0.001
Age, year, means (SD)	70.22 (11.08)	72.57 (8.70)	<0.001
Oxfordshire Community Stroke Project classification, *n* (%)			0.374
Partial anterior circulation infarct	309 (60.0)	254 (59.5)	
Total anterior circulation infarct	92 (17.9)	87 (20.4)	
Lacunar infarct	9 (1.7)	12 (2.8)	
Posterior circulation infarct	105 (20.4)	74 (17.3)	
Stroke severity, *n* (%)			<0.001
Mild	241 (46.5)	132 (30.5)	<0.001
Moderate	124 (23.9)	133 (30.7)	0.019
Severe	153 (29.5)	168 (38.8)	0.003
Neurological function deficit: median (interquartile range)			
National Institutes of Health Stroke Scale	9 (14)	13 (15)	<0.001
Barthel index	40 (60)	20 (50)	<0.001
Modified Rankin Scale	4 (3)	4 (2)	<0.001
Receiving oral anticoagulants, *n* (%)	396 (76.4)	333 (76.9)	0.912
Laboratory examination (mmol/L)			
Total cholesterol	4.56 (0.99)	5.27 (1.33)	<0.001
Triglyceride	1.28 (0.91)	1.36 (1.01)	0.211
High-density lipoprotein cholesterol	1.08 (0.28)	1.24 (0.41)	<0.001
Low-density lipoprotein cholesterol	2.83 (0.83)	3.27 (1.06)	<0.001
Fasting glucose	6.46 (2.46)	6.76 (3.09)	0.133
Glycosylated hemoglobin	6.39 (1.24)	6.23 (0.85)	0.165

### Sex Differences in Risk Factors for Stroke in Patients with NVAF

Table 2 shows that there were higher prevalences of hypertension, dyslipidemia, and obesity in women than in men, but the reverse trends were found with respect to current smoking and alcohol consumption. No significant sex differences in the prevalence of DM and/or arterial stenosis were observed in this study. In stroke survivors without NVAF, there were different results. Women were more likely than men to have DM, but men were more likely than women to have arterial stenosis (Table S1 in Supplementary Material).

**Table 2:** **Sex differences in risk factors of acute ischemic stroke with atrial fibrillation**.

Risk factors	Men *n* (%)	Women *n* (%)	*P*
Hypertension	317 (61.2)	299 (69.1)	0.012
Diabetes	124 (23.9)	114 (26.3)	0.397
Dyslipidemias	107 (20.7)	129 (29.8)	0.001
Obesity	60 (11.6)	80 (18.5)	0.003
Artery stenosis	97 (18.7)	75 (17.3)	0.575
Current smoking	174 (33.6)	53 (12.2)	<0.001
Alcohol consumption	72 (13.9)	4 (0.9)	<0.001

### Sex Differences in Outcomes after Stroke in Patients with NVAF

Dependency and recurrence rates within 36 months after stroke were 64% and 1.03-fold higher in women than in men, respectively, after adjustment for clinical features and risk factors (Table 3). Moreover, non-stroke-related mortality was 3.4% in men and 4.6% in women at 3 months, 3.6% in men and 1.8% in women at 12 months, and 4.1% in men and 4.0% in women at 36 months (all *P* > 0.05). However, there were no sex differences in outcomes among individuals with stroke without AF after adjusting for covariates, although a significantly increased risk of mortality, recurrence, and dependency were observed in women in the univariate analysis (Table S2 in Supplementary Material).

**Table 3:** **Sex differences in outcome after stroke within 3, 12, and 36 months among acute ischemic stroke patients with atrial fibrillation**.

Outcomes	Men *n* (%)	Women *n* (%)	Unadjusted		Adjusted	
			OR (95% CI)	*P*	OR (95% CI)	*P*
**3 months**						
Mortality	84 (16.6)	70 (16.7)	1.01 (0.71, 1.42)	0.976	–	–
Dependency	133 (26.3)	136 (32.5)	1.34 (1.01, 1.79)	0.042	1.09 (0.80, 1.48)	0.598
Recurrence	25 (6.0)	17 (4.9)	0.81 (0.43, 1.53)	0.519	–	–
**12 months**						
Mortality	128 (27.7)	88 (23.2)	0.79 (0.58, 1.08)	0.139	–	–
Dependency	225 (48.7)	225 (59.4)	1.54 (1.17, 2.03)	0.002	1.12 (0.82, 1.51)	0.477
Recurrence	72 (21.7)	71 (24.5)	1.17 (0.81, 1.70)	0.409	–	–
**36 months**						
Mortality	128 (36.3)	121 (43.7)	1.36 (0.99, 1.88)	0.059	–	–
Dependency	267 (75.6)	238 (85.9)	1.97 (1.30, 2.98)	0.001	1.64 (1.02, 2.64)	0.043
Recurrence	106 (48.8)	106 (68.8)	2.31 (1.50, 3.56)	<0.001	2.03 (1.28, 3.20)	0.002

Moreover, we performed a multivariate analysis to assess the determinants of stroke outcomes among patients with NVAF. We found that, except for age, OCSP classification, and stroke severity, the determinants of mortality were obesity at 3 months, drinking alcohol at 12 and 36 months, and DM at 36 months. For recurrence, drinking alcohol was an independent determinant at 12 and 36 months; in addition, hypertension was significantly associated with recurrence risk at 12 months. However, age and female sex were independent risk factors of recurrence at 36 months. Age, stroke severity, and drinking alcohol were associated with the risk of dependency at 3, 12, and 36 months; OCSP classification was associated with the risk of dependency at 3 months; hypertension was associated with the risk of dependency at 12 months; and female and obesity were associated with the risk of dependency at 36 months (Tables S3–S5 in Supplementary Material).

## Discussion

To our knowledge, this is the first report of the sex differences in long-term outcomes among stroke survivors with AF. In this study, we assessed the sex differences in clinical features, medical histories, and outcomes at 3, 12, and 36 months after stroke in patients with NVAF.

Rates of AF vary significantly among different racial and ethnic groups (31, 32). Hispanics and Asians have lower rates of AF than do non-Hispanic whites (32–37). A similar lower prevalence of AF in the general population was reported in China (35–37). The difference in rates of AF among race-ethnicity groups may be explained by differences in underlying genetic susceptibility, differences in the prevalence of risk factors for AF, differences in the hazards due to individual risk factors, or a combination of these variables (38).

Women had a 14–70% increased risk of stroke compared to that in men. The results of a multivariable analysis from the anticoagulation and risk factors in atrial fibrillation (ATRIA) study cohort indicated that women had higher annual rates of thromboembolism off warfarin than did men [relative risk (RR) = 1.6; 95% CI: 1.3–1.9] (11). Women developed primary events more often (2.08%/year, 95% CI: 1.60–2.56 vs. 1.44%/year, 95% CI: 1.18–1.71%/year in men; *P* = 0.016) (13). There was a higher risk of stroke in anticoagulated AF women than in men, despite a similar anticoagulation RR for women compared to men 2.0 (95% CI: 1.3–3.1; *P* = 0.004) in Italy (14). A population-based cohort study from Canada revealed that women had a higher risk of stroke than men did (adjusted hazard ratio = 1.14; 95% CI: 1.07–1.22; *P* < 0.001), even after adjusting for baseline comorbid conditions, individual components of the CHADS_2_ score, and warfarin treatment (39). Similar trends was observed in the Framingham Heart Study, after warfarin censoring and adjustment for age and systolic blood pressure, women had a higher risk of stroke than men did (adjusted hazard ratio = 1.73; 95% CI: 1.16–2.59) (40). Moreover, a higher prevalence of AF in women among stroke survivors has been frequently reported (6, 15, 41–43). A hospital-based study from China reported a higher prevalence of AF in women than in men, with a prevalence of 19.4% in women and 10.6% in men (41). Another study conducted in Israel indicated that 19.6% of stroke patients had AF, representing 27.1% in women and 13.6% in men (42). The European Community Stroke Project revealed that the prevalence of stroke with AF was 20.8% in women and 15.2% in men (43). The North Dublin Population Stroke Study, a population-based prospective cohort study, observed a rate of 38.1% in women and 31.9% in men (6). Similar results have been reported in another population-based study in Italy (25). Consistent with these studies, we observed a 64% increased frequency of AF in women with stroke than in men with stroke. A higher risk for stroke in women with AF can explain the higher frequency of AF in women with stroke than in men with stroke (8, 44).

Previous studies reported that women were more likely than men to be older (6, 15, 24, 41–43) and to have severe stroke and poor neurological function on admission (6, 24, 41, 43). Women have a greater number of stroke risk factors, such as DM, hypertension, chronic renal disease, cardiovascular disease, and heart failure, than men do at baseline (8, 11–13, 39). Consistent with these studies, we found that women were 2 years younger than men at stroke onset. Women were more likely to have hypertension, dyslipidemia, and obesity, but there appeared to be no sex differences in the prevalence of DM or arterial stenosis in the present study. Higher levels of TC, HDL-C, and LDL-C occurred in women, but men were more likely to be current smokers and alcohol consumers. Reduced or non-standard management of risk factors (including hypertension and dyslipidemia) due to one’s culture and economic status may partly explain the sex differences in the frequency of risk factors in this study.

The impact of sex on poor stroke outcomes in patients with AF is not clear. A study has suggested that sex was not associated with increased mortality when age and medical comorbidities were controlled (45). Similarly, the Framingham Heart Study did not observe a significant difference in the mortality rate between women and men (46). In accordance with this study, sex differences in mortality after stroke was not observed among NVAF patients with stroke in this study.

Findings related to the effects of AF on recurrence and dependency in individuals with stroke have been inconsistent. A few studies have indicated a greater recurrence rate and a markedly increased dependency rate in stroke patients with AF (22, 47, 48).

The evidence regarding discrepancies in stroke outcomes among patients with AF between men and women with AF is inconsistent. A few studies demonstrated that women had a higher rate of poor outcomes for individuals with ischemic stroke and AF (39, 49). It has been reported that women had worse functional outcomes (defined as an mRS ≥3) at 3 months after stroke due to AF (50). However, this report was in contrast with the result of another study, which reported a higher frequency of independency at 1 year after stroke related to AF in men than in women (25). In the present study, the rates of dependency and recurrence at 36 months after stroke were significantly higher in women than in men after adjustment for age, stroke severity, and conventional risk factors for stroke; the long-term risks of dependency and recurrence in women were increased by 84% and 1.18-fold, respectively. Furthermore, women were more likely to have hypertension, dyslipidemia, and obesity in this study. These factors may have contributed to the poor outcomes for women with AF and stroke. In addition, non-standard management of AF in women due to their low social and economic status can explain partly the poorer stroke outcomes in women than in men.

There were some limitations to this study. The first is the limited representativeness of patients included, because data were included only for patients from three hospitals in the same city. The second is the lack of data regarding any prescribed medication therapy prior to stroke occurrence, including traditional medications; especially lack of information of oral anticoagulants previously. These may result in bias of evaluation the effected factors for stroke outcomes. Third, differences in the rehabilitation treatment and technique during the study period may have affected the result. However, the aim in this study, which was to assess sex differences in the clinical features and outcomes among stroke survivors with AF, may reduce the effect. Finally, there may be a measurement bias in NIHSS, BI, and mRS assessments, because some patients were assessed in person, whereas others were assessed over the phone.

## Conclusion

In this large, hospital-based stroke registry study, we assessed sex differences in clinical features, risk factors, and short- and long-term outcomes following stroke in patients with AF. There was a higher prevalence of AF, severe stroke, hypertension, dyslipidemia, and obesity and an older age at stroke onset in women than in men, but men were more likely to be current smokers and alcohol consumers. However, in those patients without AF, the frequency of PACI was higher in women than in men, but the frequency of POCI was higher in men than in women. Women were observed to have poorer long-term outcomes, including higher risks of dependency and recurrence. These findings suggest that it is crucial to emphasize the need for individualized stroke prevention education and promotion of healthy lifestyles in order to improve AF-related stroke outcomes and reduce disease burden in women.

## Ethics Statement

This study was carried out in accordance with the recommendations of international ethical guidelines for biomedical research involving human subjects (51), and the ethics committee of Tianjin Huanhu Hospital and Tianjin Medical University General Hospital approved this study. All subjects provided written informed consent, in accordance with the Declaration of Helsinki.

## Author Contributions

ZA, JW, and XN contributed to the design, data collection, data interpretation, and critical review. YH contributed to draft the manuscript. JW contributed to data analysis. YH, XY, WZ, XZ, JZ, YY, and ZA contributed to data collection, case diagnosis, and confirmation.

## Conflict of Interest Statement

The authors declare that the research was conducted in the absence of any commercial or financial relationships that could be construed as a potential conflict of interest.
