# Supplementary material for: Sex Differences in Outcomes among Stroke Survivors with Non-Valvular Atrial Fibrillation in China
Source: Front Neurol. 2017 Apr 27;8:166. doi: 10.3389/fneur.2017.00166 (PMC5406396; doi:10.3389/fneur.2017.00166)
Supplement: Supplementary file 1 [file Table_1.DOCX]

Supplemental Table 1. Sex Differences in clinical and demographical characteristics in ischemic stroke patients with AF.

| Characteristics | Men | Women | P |
| --- | --- | --- | --- |
| Age, year, means(SD) | 62.11 (11.61) | 66.20 (10.89) | < 0.001 |
| OCSP classification, n (%): |  |  | 0.001 |
| PACI | 3636 (52.5) | 1890 (56.6) | < 0.001 |
| TACI | 307 (4.4) | 139 (4.2) | 0.454 |
| LACI | 499 (7.2) | 236 (7.1) | 0.791 |
| POCI | 2479 (35.8) | 1075 (32.2) | < 0.001 |
| Stroke severity, n (%): |  |  | < 0.001 |
| Mild | 4807 (68.7) | 2150 (63.7) | < 0.001 |
| Moderate | 1669 (23.9) | 901 (26.7) | 0.002 |
| Severe | 519 (7.4) | 326 (9.7) | < 0.001 |
| Neurological function deficit: median (interquartile range) |  |  |  |
| NIHSS | 5 (7) | 6 (7) | < 0.001 |
| BI | 60 (50) | 55 (50) | < 0.001 |
| mRS | 3 (2) | 4 (2) | < 0.001 |
| Receiving oral anticoagulants, n (%) | 568 (8.2) | 251 (7.6) | 0.288 |
| Laboratory examination: mmol/L |  |  |  |
| Total cholesterol | 4.75 (1.03) | 5.29 (1.12) | < 0.001 |
| Triglyceride | 1.60 (1.01) | 1.73 (1.36) | < 0.001 |
| High density lipoprotein cholesterol | 1.02 (0.37) | 1.14 (0.29) | < 0.001 |
| Low density lipoprotein cholesterol | 2.95 (0.82) | 3.22 (0.89) | < 0.001 |
| Fasting glucose | 6.44 (2.66) | 6.79 (2.77) | < 0.001 |
| Glycosylated hemoglobin | 6.49 (1.82) | 6.83 (1.56) | < 0.001 |
| Risk factors, n (%) |  |  |  |
| Hypertension | 4950 (70.7) | 2680 (79.2) | < 0.001 |
| Diabeyes | 2099 (30.0) | 1278 (37.8) | < 0.001 |
| Dyslipidemias | 2042 (29.2) | 1187 (35.1) | < 0.001 |
| Artery stenosis | 1785 (25.5) | 703 (20.8) | < 0.001 |
| Obesity | 622 (8.9) | 676 (20.0) | < 0.001 |
| Smoking | 3687 (12.7) | 480 (14.2) | < 0.001 |
| Alcohol consumption | 1980 (28.3) | 38 (1.1) | < 0.001 |
