# Supplementary material for: Sex Differences in Outcomes among Stroke Survivors with Non-Valvular Atrial Fibrillation in China
Source: Front Neurol. 2017 Apr 27;8:166. doi: 10.3389/fneur.2017.00166 (PMC5406396; doi:10.3389/fneur.2017.00166)
Supplement: Supplementary file 2 [file Table_2.DOCX]

Supplemental Table 2. Sex differences in outcome after stroke within 3, 12, and 36 months among acute ischemic stroke patients with AF.

| Outcomes | Men | Women | Unadjusted | |  | Adjusted | |
| --- | --- | --- | --- | --- | --- | --- | --- |
|  |  |  | OR(95%CI) | P |  | OR(95%CI) | P |
| 3 months: |  |  |  |  |  | — | — |
| Mortality | 379 (5.5) | 195 (5.9) | 1.07 (0.89, 1.28) | 0.471 |  | — | — |
| Recurrence | 366 (4.1) | 107 (3.4) | 0.83 (0.66, 1.04) | 0.111 |  | — | — |
| Dependency | 909 (13.2) | 469 (14.1) | 1.08 (0.96, 1.22) | 0.223 |  | — | — |
| 12 months: |  |  |  |  |  |  |  |
| Mortality | 501 (8.1) | 293 (9.8) | 1.24 (1.06, 1.44) | 0.006 |  | 0.88 (0.73, 1.06) | 0.172 |
| Recurrence | 786 (13.8) | 392 (14.6) | 1.07 (0.94, 1.22) | 0.309 |  | — | — |
| Dependency | 1823 (29.5) | 1001 (33.6) | 1.21 (1.10, 1.33) | < 0.001 |  | 0.95 (0.85, 1.06) | 0.351 |
| 36 months: |  |  |  |  |  |  |  |
| Mortality | 645 (14.9) | 390 (18.5) | 1.30 (1.13, 1.49) | < 0.001 |  | 0.97 (0.82, 1.15) | 0.754 |
| Recurrence | 1247 (35.5) | 689 (41.5) | 1.29 (1.15, 1.46) | < 0.001 |  | 0.98 (0.86, 1.13) | 0.790 |
| Dependency | 2489 (57.5) | 1277 (60.7) | 1.14 (1.03, 1.27) | 0.013 |  | 0.89 (0.79, 1.01) | 0.076 |
