# Supplementary material for: Sex Differences in Outcomes among Stroke Survivors with Non-Valvular Atrial Fibrillation in China
Source: Front Neurol. 2017 Apr 27;8:166. doi: 10.3389/fneur.2017.00166 (PMC5406396; doi:10.3389/fneur.2017.00166)
Supplement: Supplementary file 3 [file Table_3.DOCX]

Supplemental Table 3. Determinants of outcomes at 3 months after stroke among patients with NVAF.

| Risk Factors | Reference | Mortality | |  | Recurrence | |  | Dependency | |
| --- | --- | --- | --- | --- | --- | --- | --- | --- | --- |
|  |  | OR (95%CI) | P |  | OR (95%CI) | P |  | OR (95%CI) | P |
| Women | Men | — | — |  | — | — |  | — | — |
| Age | — | 1.02 (1.00, 1.04) | 0.029 |  | — | — |  | 1.02 (1.00, 1.03) | 0.026 |
| OCSP: | POCI |  |  |  |  |  |  |  |  |
| PACI |  | 0.72 (0.41, 1.27) | 0.258 |  | — | — |  | 0.79 (0.53, 1.19) | 0.259 |
| TACI |  | 2.20 (1.19, 4.08) | 0.012 |  | — | — |  | 2.17 (1.32, 3.58) | 0.002 |
| LACI |  | — | 0.998 |  | — | — |  | — | 0.998 |
| Severity: | Mild |  |  |  |  |  |  |  |  |
| Moderate |  | 2.88 (1.54, 5.38) | 0.001 |  | — | — |  | 1.37 (0.91, 2.06) | 0.134 |
| Severe |  | 7.12 (3.99, 12.72) | <0.001 |  | — | — |  | 2.39 (1.62, 3.54) | <0.001 |
| Hypertension | No | — | — |  | — | — |  | — | — |
| Diabetes | No | — | — |  | — | — |  | — | — |
| Dyslipidemia | No | — | — |  | — | — |  | — | — |
| Obesity | No | 0.37 (0.19, 0.71) | 0.003 |  | — | — |  | — | — |
| Alcohol consumption | No | — | — |  | — | — |  | 0.49 (0.25, 0.99) | 0.045 |
