# Supplementary material for: Sex Differences in Outcomes among Stroke Survivors with Non-Valvular Atrial Fibrillation in China
Source: Front Neurol. 2017 Apr 27;8:166. doi: 10.3389/fneur.2017.00166 (PMC5406396; doi:10.3389/fneur.2017.00166)
Supplement: Supplementary file 4 [file Table_4.DOCX]

Supplemental Table 4. Determinants of outcomes at 12 months after stroke among patients with NVAF.

| Risk Factors | Reference | Mortality | |  | Recurrence | |  | Dependency | |
| --- | --- | --- | --- | --- | --- | --- | --- | --- | --- |
|  |  | OR (95%CI) | P |  | OR (95%CI) | P |  | OR (95%CI) | P |
| Women | Men | — | — |  | — | — |  | — | — |
| Age | — | 1.04 (1.02, 1.05) | <0.001 |  | — | — |  | 1.03 (1.02, 1.05) | <0.001 |
| OCSP: | POCI |  |  |  |  |  |  |  |  |
| PACI |  | 0.48 (0.30, 0.78) | 0.003 |  | — | — |  | — | — |
| TACI |  | 1.13 (0.65, 1.97) | 0.670 |  | — | — |  | — | — |
| LACI |  | — | 0.998 |  | — | — |  | — | — |
| Severity: | Mild |  |  |  |  |  |  |  |  |
| Moderate |  | 3.16 (1.88, 5.30) | <0.001 |  | — | — |  | 1.64 (1.13, 2.38) | 0.009 |
| Severe |  | 7.79 (4.72, 12.86) | <0.001 |  | — | — |  | 3.46 (2.35, 5.10) | <0.001 |
| Hypertension | No | — | — |  | 1.58 (1.05, 2.38) | 0.027 |  | 1.42 (1.04, 1.92) | 0.026 |
| Diabetes | No | — | — |  | — | — |  | — | — |
| Dyslipidemia | No | — | — |  | — | — |  | — | — |
| Obesity | No | — | — |  | — | — |  | — | — |
| Alcohol consumption | No | 0.40 (0.17, 0.96) | 0.039 |  | 0.27 (0.11, 0.70) | 0.007 |  | 0.32 (0.17, 0.61) | <0.001 |
