# Supplementary material for: Sex Differences in Outcomes among Stroke Survivors with Non-Valvular Atrial Fibrillation in China
Source: Front Neurol. 2017 Apr 27;8:166. doi: 10.3389/fneur.2017.00166 (PMC5406396; doi:10.3389/fneur.2017.00166)
Supplement: Supplementary file 5 [file Table_5.DOCX]

Supplemental Table 5. Determinants of outcomes at 36 months after stroke among patients with NVAF.

| Risk Factors | Reference | Mortality | |  | Recurrence | |  | Dependency | |
| --- | --- | --- | --- | --- | --- | --- | --- | --- | --- |
|  |  | OR (95%CI) | P |  | OR (95%CI) | P |  | OR (95%CI) | P |
| Women | Men | — | — |  | 2.03 (1.28, 3.20) | 0.002 |  | 1.64 (1.03, 2.64) | 0.043 |
| Age | — | 1.07 (1.04, 1.09) | <0.001 |  | 1.02 (1.00, 1.05) | 0.023 |  | 1.06 (1.04, 1.08) | <0.001 |
| OCSP: | POCI |  |  |  |  |  |  |  |  |
| PACI |  | 0.45 (0.27, 0.75) | 0.002 |  | — | — |  | — | — |
| TACI |  | 0.81 (0.43, 1.54) | 0.524 |  | — | — |  | — | — |
| LACI |  | — | 0.998 |  | — | — |  | — | — |
| Severity: | Mild |  |  |  |  |  |  |  |  |
| Moderate |  | 3.26 (1.95, 5.44) | <0.001 |  | — | — |  | 1.94 (1.14, 3.30) | 0.043 |
| Severe |  | 8.23 (4.93, 13.74) | <0.001 |  | — | — |  | 4.17 (2.40, 7.25) | <0.001 |
| Hypertension | No | — | — |  | — | — |  | 2.74 (1.75, 4.29) | <0.001 |
| Diabetes | No | 1.95 (1.25, 3.06) | 0.003 |  | — | — |  | — | — |
| Dyslipidemia | No | — | — |  | — | — |  | — | — |
| Obesity | No | — | — |  | — | — |  | 0.37 (0.21, 0.67) | 0.001 |
| Alcohol consumption | No | 0.42 (0.17, 1.00) | 0.050 |  | 0.41 (0.19, 0.89) | 0.024 |  | 0.43 (0.21, 0.89) | 0.023 |
